# The Brown-Vialetto-Van Laere and Fazio Londe syndrome revisited: natural history, genetics, treatment and future perspectives

**DOI:** 10.1186/1750-1172-7-83

**Published:** 2012-10-29

**Authors:** Annet M Bosch, Kevin Stroek, Nico G Abeling, Hans R Waterham, Lodewijk IJlst, Ronald JA Wanders

**Affiliations:** 1 The Department of Pediatrics, University of Amsterdam, Amsterdam, The Netherlands; 2 Laboratory Genetic Metabolic Diseases, Academic Medical Center, University of Amsterdam, Amsterdam, The Netherlands

**Keywords:** Brown-Vialetto-Van Laere syndrome, Fazio Londe syndrome, Riboflavin transporter, Riboflavin supplementation

## Abstract

The Brown-Vialetto-Van Laere syndrome is a rare neurological disorder which may present at all ages with sensorineural deafness, bulbar palsy and respiratory compromise. Fazio-Londe syndrome is considered to be the same disease entity. Recently it was demonstrated that in some patients the disease is caused by mutations in the *SLC52A3* gene which encodes the intestinal (hRFT2) riboflavin transporter. In these patients riboflavin deficiency is the cause of the BVVL/FL syndrome and supplementation of riboflavin proved a life saving treatment. Mutations in the *SLC52A2* gene and the *SLC52A1 (GPR172B)* gene, coding for human riboflavin transporters hRFT3 and hRFT1 have been associated with the BVVL syndrome as well. We performed a review of the literature, with emphasis on the natural history and the effects of treatment in these patients**.** A total of 35 publications were traced reporting on the clinical presentation of 74 patients who presented before age 18. The most prevalent symptoms were bulbar palsy, hearing loss, facial weakness and respiratory compromise. Death was reported in 28 of the 61 untreated patients, with a very low survival in patients presenting before age 4. All 13 patients who were treated with riboflavin survived, with a strong clinical improvement after days to months of treatment in eight patients. Three patients demonstrated a stable clinical course and treatment was stopped early in two patients. Abnormalities in plasma flavin levels and/or plasma acylcarnitine profiles were observed in some but not in all patients, and also patients with normal plasma flavin levels and acylcarnitine profiles demonstrated a striking clinical improvement on riboflavin supplementation. It is now clear that proper diagnosis requires mutation analysis of all three transporter genes and treatment should be started immediately without first awaiting results of molecular analysis. Clinical improvement may be rapid or gradual over a period of more than 12 months.

## Introduction

The Brown-Vialetto-Van Laere syndrome (BVVL) (MIM 211530, ORPHA97229) is a rare neurological disorder first described by Brown in 1894 and later by Vialetto and Van Laere. The prevalence is very low and only fifty-eight patients were reported by 2008 [1]. Patients mostly present with sensorineural deafness, bulbar palsy and respiratory compromise and the age of onset varies from infancy to adulthood [1]. In Fazio-Londe syndrome (FL) (MIM 211500, ORPHA56965) the clinical presentation is the same, but without the hearing loss, and FL is considered to be the same disease entity as BVVL [2]. Bosch et al. [3] demonstrated the same homozygous mutation in two siblings who presented with BVVL and FL syndromes respectively [3].

In 2008 a detailed review of the literature was published by Sathasivam in this journal [1]. However, since 2010 insights into the pathophysiology, genetics and treatment of this disorder have increased tremendously. In 2010 Green et al. demonstrated that in some patients the disease is caused by mutations in the *SLC52A3* gene (other aliases: *BVVLS, BVVLS1, C20orf54, RFT2, RFVT3, bA371L19.1, hRFT2*) [4], and in 2011 Bosch et al. demonstrated that this gene encodes the intestinal (hRFT2) riboflavin transporter and that in these patients riboflavin deficiency is the cause of the BVVL/FL syndrome [3]. Supplementation of riboflavin proved a life saving treatment for a number of young patients [3-7]. Inheritance is autosomal recessive [3,4].

Recently, mutations in the *SLC52A2* gene (other aliases: *BVVLS2, D15Ertd747e, GPCR41, GPR172A, PAR1, RFT3, RFVT2, hRFT3*) coding for another human riboflavin transporter (hRFT3) have been associated with the BVVL syndrome as well, again with favorable clinical effects of oral riboflavin supplementation [8,9]. Furthermore, Ho et al. [10] reported a newborn who presented with clinical features highly suggestive of multiple acyl-CoA dehydrogenase deficiency (MADD) resulting from maternal riboflavin deficiency [10]. The mother was was found to have a hemizygous deletion spanning exons 2 and 3 of the *SLC52A1* gene (other aliases: *GPCR42, GPR172B, PAR2, RFT1, RFVT1, hRFT1*) which encodes another riboflavin transporter. The infant fully recovered after riboflavin treatment [10,11].

Awareness of these new insights and therapeutic options among professionals is essential, especially since treatment appears to be lifesaving with obvious consequences for the outcomes of these patients. Therefore, we performed a review of the literature, with particular emphasis on the natural history and the effects of treatment in these patients, and provide advice on diagnostics and treatment based on the present knowledge.

## Methods

### Literature search

A search was done in Pubmed using the search terms ‘Brown-Vialetto-Van Laere’ and ‘Fazio-Londe’. As these disorders are considered to be the same disease entity we will report all patients as having BVVL syndrome in this article [2,3]. We searched for publications in English. Every accessible publication published before September 1st 2012 was studied for case reports. All case reports were carefully analyzed for clinical aspects such as age at presentation, presenting symptoms, course of the disease, survival, treatment and genetics. Only patients who presented before the age of 18 were included and all patients described more than once were included only once. Case reports without sufficient information about age or symptoms were excluded. References were checked for other relevant publications, which did not result in additional publications since no additional patients were described in these publications. An additional search in EBSCO Medline gave no further relevant results.

## Results

A total of 34 accessible publications was retrieved. Of one more article only the abstract was available but this provided enough information to be useful [12]. In these 35 publications 101 patients are described. After excluding doubles (10) and patients with a presentation at age 18 or above or no information about age (17), the data of 74 patients were analyzed [2-9,12-30].

### Patients

**Figure 1:**
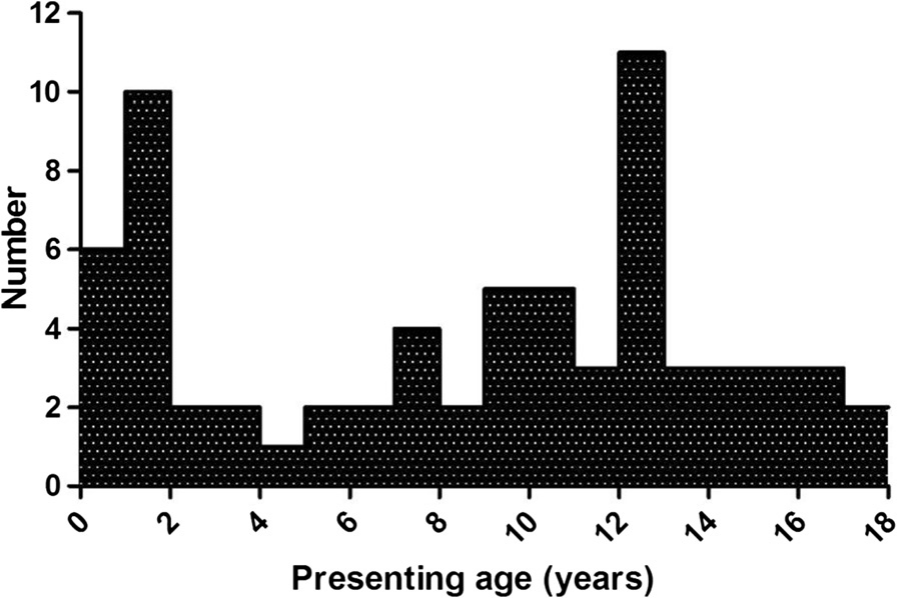
Number of patients by age of presentation.

Seventy-four case reports were analyzed. The group consisted of 23 males and 51 females. Because the incidence within this group varied with age, we decided to divide the patients in three age groups (Figure 1). Group 1 contains 21 patients with an age at presentation between 0 and 3 years. One of these patients was reported to present in infancy without an exact age. For this reason she was excluded from the group in which the presenting age was studied [28]. Group 2 contains 21 patients with a presenting age range of 4 through 10 years and group 3 contains 28 patients with a presenting age range of 11 through 17 years. Because four other patients were reported as “presenting in childhood”, they could not be included in one particular group, but were included when we report on data of the complete patient group [14,20,24,30]. 

## Natural history

### Presenting age

The age at presentation was known for 69 patients with a mean of 8.2 years (median 9, range 0.3-17, SD 5.2). In group 1 (N=21) the age at presentation was known for 20 patients and the mean presenting age was 1.4 years. In group 2 (N=21) and in group 3 (N=28) the mean presenting ages were 7.9 years and 13.3 years respectively (Table 1).

**Table 1:** Presenting age in years of all patients

**Presenting age (years)**	**Mean**	**Median**	**Age range**	**SD**
Group 1* (N=20)	1.4	1.3	0.3 – 3.2	0.8
Group 2* (N=21)	7.9	8.0	4 – 10	1.8
Group 3* (N=28)	13.3	12.5	11 – 17	1.8
All patients (N=69)	8.2	9.0	0.3 – 17	5.2

*Group 1: age <4 years, Group 2: age 4–10 years, Group 3: age 11–17 years.

### Clinical presentation

Symptoms of a total of 73 patients were reported. For one patient symptoms were not specified [24]. For the complete group the most prevalent symptoms were bulbar palsy (92%), hearing loss (81%), facial weakness (77%) and respiratory compromise (64%). In group 1 (N=21) the most prevalent symptoms were respiratory compromise and bulbar palsy (both 86%). Muscle weakness and hearing loss were found in 67% of patients. In all patients included in group 2 (N=21) bulbar palsy was found, with facial weakness in 95%, hearing loss in 90% and respiratory compromise in 62% of patients. In group 3 (N=28) bulbar palsy (89%), hearing loss (86%), and facial weakness (82%) were prevalent, with muscle weakness in 50% of patients (Table 2). 

**Table 2:** Group 1, 2 and 3 symptoms

	**Group 1 (N=21)**	**%**	**Group 2 (N=21)**	**%**	**Group 3 (N=28)**	**%**	**Total (N=73)**	**%**
Bulbar palsy (CN IX-XII)	18	86%	21	100%	25	89%	67	92%
Hearing loss	14	67%	19	90%	24	86%	59	81%
Facial weakness	10	48%	20	95%	23	82%	56	77%
Respiratory compromise	18	86%	13	62%	13	46%	47	64%
Muscle weakness	14	67%	10	48%	14	50%	40	55%
Upper motor neuron signs	3	14%	10	48%	11	39%	26	36%
Palsy of CN III, IV or VI	9	43%	5	24%	7	25%	23	32%
Stridor	10	48%	3	14%	4	14%	18	25%
Palsy of the trigeminal nerve (V)	1	5%	6	29%	6	21%	14	19%
Lower motor neuron signs	4	19%	5	24%	4	14%	13	18%
Diaphragm weakness/palsy	4	19%	3	14%	4	14%	11	15%
Palsy of the optic nerve (II)	2	10%	3	14%	5	18%	10	14%
Ataxia	2	10%	3	14%	3	11%	9	12%
Tremor	2	10%	1	5%	3	11%	6	8%
Behavioral changes	1	5%	0	0%	2	7%	3	4%
Mental retardation	0	0%	0	0%	3	11%	3	4%
Epilepsy	0	0%	0	0%	2	7%	3	4%
Autonomic dysfunction	0	0%	0	0%	2	7%	2	3%

### Survival

For this section patients who received riboflavin treatment were analyzed separately from the untreated patients. All 13 patients who received treatment survived [3,5-9].

In the 61 patients who did not receive riboflavin treatment death was reported in 28 of the patients (Table 3). Mean age of death was 11.6 years (median 9.3, range 0.9–42, SD 11.1). The mean time span between presentation and death was specified for 25 patients and was 5.0 years (median 1.3, range 0–32, SD 7.9). The patients were divided in three groups by the same age ranges as described earlier. In the untreated patients in group 1 (N=13), death was reported in 11 patients. Mean age of death was 1.9 years (median: 1.9; range: 0.9–3; SD: 0.6). The mean time span between presentation and death was 0.8 years (median: 0.6; range: 0.2–1.7; SD: 0.5). In 6 patients the cause of death was known and in all of them respiratory insufficiency was reported as the cause. In group 2 (N=17) 9 patients did not survive. Mean age of death was 18.5 years (median: 11; range: 10.5–42; SD: 11.0). The mean time span between presentation and death was 10.3 years (median: 3.5; range: 0.3–32; SD: 10.7). In 4 patients the cause of death was known and respiratory insufficiency was reported as cause of death in all 4 patients. In group 3 (N=27) death was reported in 5 cases. Mean age of death was 18.5 years (median: 17.3; range: 12–25; SD: 4.6). The mean time span between presentation and death was 4.7 years (median: 5.3; range: 0–10; SD: 4.1). In 2 patients the cause of death was known and in both of them respiratory insufficiency was reported as the cause.

**Table 3:** Age of death in years in patients who did not receive riboflavin treatment

**Age of death (years)**	**N deceased**	**% deceased**	**Mean age**	**Median age**	**Age range**	**SD**
Group 1* (N=13)	11	85%	1.9	1.9	0.9 – 3	0.6
Group 2* (N=17)	9	53%	18.5	11	7.3 – 42	11.0
Group 3* (N=27)	5	19%	18.5	17.3	12 – 25	4.6
All patients (N=61)	28	46%	11.6	9.3	0.9 – 42	11.1

*Group 1: age <4 years, Group 2: age 4–10 years, Group 3: age 11–17 years.

## Genetics

Until now, 23 different mutations have been reported in the *SLC52A3* gene in 23 patients included in this review [3,4,6,7,9,13,20] (Table 4). Anand et al. [5] reported a pathogenic homozygous mutation in their patient without providing details on the mutation [5]. The mutations include frameshift, splice site, missense and nonsense mutations. By screening a cohort of patient suspected of BVVL syndrome Johnson et al. [9] demonstrated two more mutations in patients not included in the natural history review because clinical symptoms were not reported [9]. These two mutations are included in Table 4. 

**Table 4:** **Reported pathogenic mutations in the*SLC52A3*gene**

**A Reported pathogenic mutations in the*SLC52A3*gene**				
**Mutation**	**Type**	**Exon**	**Coding effect**	**Reference (first report)**
c.49T>C	Missense	2	p.Trp17Arg	Bosch et al. [3]
c.62A>G	Missense	2	p.Asn21Ser	Dezfouli et al. [13]
c.82C>A	Missense	2	p.Pro28Thr	Johnson [20]
c.106G>A^1^	Missense	2	p.Glu36Lys	Green et al. [4]
c.160G>A	Missense	2	p.Gly54Arg	Johnson [9]
c.173T>A	Missense	2	p.Val58Asp	Ciccolella et al. [6]
c.211G>A	Missense	2	p.Glu71Lys	Johnson. [20]
c.211G>T	Nonsense	2	p.Glu71X	Green et al. [4]
c.224T>C	Missense	2	p.Ile75Thr	Johnson [9]
c.394C>T	Missense	2	p.Arg132Trp	Green et al. [4]
c.568-19_-18insCTGATTGAC	Insertion	2i	Unknown	Ciccolella et al. [6]
c.639C>G	Nonsense	3	p.Tyr213X	Green et al. [4]
c.659C>A	Missense	3	p.Pro220His	Dezfouli et al. [13]
c.670T>C	Missense	3	p.Phe224Leu	Green et al. [4]
c.796C>T	Missense	3	p.Arg266Trp	Ciccolella et al. [6]
c.935C>T	Missense	3	p.Ala312Val	Dezfouli et al. [13]
c.955C>T	Missense	3	p.Pro319Ser	Ciccolella et al. [6]
c.989G>T	Missense	3	p.Gly330Val	Koy et al. [7]
c.1048T>A	Missense	3	p.Leu350Met	Green et al. [4]
c.1325_1326delTG	Deletion	5	p.Leu442ArgfsX64	Green et al. [4]
c.1198-2A>C	Splice defect	4i	Unknown	Bosch et al. [3]
c.1371C>G	Missense	5	p.Phe457Leu	Green et al. [4]
c.1296C>A	Nonsense	5	p.Cys432X	Ciccolella et al. [6]
c.1237T>C^1^	Missense	5	p.Val413Ala	Green et al. [4]
c.1238T>C	Missense	5	p.Val413Ala	Ciccolella et al. [6]
**B Reported pathogenic mutations in the*SLC52A2*gene**				
c.368T>C	Missense		p.Leu123Pro	Haack et al. [8]
c.419C>T^2^	Missense		p.Pro140Leu	Johnson et al. [9]
c.916G>A^2^	Missense		p.Gly306Arg	Johnson et al. [9]
c.1016T>C	Nonsense		p.Leu339Pro	Haack et al. [8]

^1^ c.106G>A and c.1237T>C are in cis. c.106G>A is predicted to be deleterious; c.1237T>C is predicted to be tolerated (Green et al. [4]).

^2^ c.419C>T is reported in cis with pathogenic mutation c.916G>A and is possibly non-pathogenic (Johnson et al. [9]).

Recently, mutations have been demonstrated in the *SLC52A2* gene which codes for hRFT3. Johnson et al. [9] demonstrated a homozygous missense mutation in two unrelated patients [9], whereas Haack et al. [8] reported the identification of two heterozygous mutations in one patient [8].

## Biochemical abnormalities

Abnormalities in the acylcarnitine profiles were reported in 6 patients, of whom 4 patients demonstrated mutations in the *SLC52A3* gene and 2 in the *SLC52A2* gene [3,5,8,9]. In 4 of these patients deficient flavin levels were found as well. All acylcarnitine profiles and flavin levels normalised after riboflavin supplementation.

## Treatment: riboflavin supplementation

The effects of supplementation of riboflavin have been reported in 13 patients [3,5-9]. Eleven patients were treated with oral riboflavin, in two patients intravenous riboflavin supplementation was reported. Most patients were treated with a dose of 10mg/kg/day. The mean age at the start of treatment was known for 11 patients and was 6.9 years (range: 3 months–17 years).

Eight of 13 treated patients demonstrated a strong clinical improvement. Seven of these patients were treated orally: one patient initially with a dose of 150 mg/day at age 10 (which will be less than 10 mg/kg/day) which was increased to 450 mg/day after 3 months, five with a dose of 10 mg/kg/day, and one patient with a dose of and 25 mg/kg/day. One patient was treated intravenously with 200 mg/day at age 9 (less than 10 mg/kg/day). The first signs of improvement were seen within days in some patients [3,5] with a more gradual improvement over many months in others [3,5,6]. Improvement was seen in muscle strength, motor function, respiration, hearing, and vision with a full recovery in some. Five patients were on mechanical ventilation (4 tracheostomy and 1 noninvasive ventilation) at the start of treatment. In all 4 patients with a tracheostomy, ventilation could be decreased or stopped. In the patient on noninvasive ventilation an improvement in diaphragm function was reported [3,5,6].

In 6 of 8 riboflavin responsive patients MADD-like abnormalities were found in the acylcarnitine profile before treatment [3,5,8,9] and in 4 of these patients deficient flavin levels were found as well. All acylcarnitine profiles and flavin levels normalised after riboflavin supplementation. Notably, 2 patients with normal acylcarnitine profiles, 3 patients with normal flavin levels, and one with unknown flavin levels before treatment was started, did demonstrate a strong clinical improvement after the start of treatment as well [6].

In all 8 patients who improved on riboflavin supplementation mutations were found in riboflavin transporter genes: in 6 patients mutations were demonstrated in the *SLC52A3* gene, whereas in 2 patients mutations were demonstrated in the *SLC52A2* gene [8,9].

In 5 patients no improvement was demonstrated with riboflavin supplementation. Two of these patients were treated for a very short period: 1 discontinued treatment because of intolerance to oral riboflavin [6], and in one patient treatment was stopped already after 1 week due to lack of clinical improvement [7]. This last patient did demonstrate improvement in the following months but the relation between the improvement and the riboflavin supplementation has remained unclear. All three patients on prolonged riboflavin supplementation are reported to have a stable clinical situation but without improvement. The three stable patients and the patient who stopped treatment because of intolerance were all treated orally with 10 mg/kg/day. The patient who was considered unresponsive after one week of treatment was treated with 10 mg/kg/day intravenously. In four of the patients without improvement, acylcarnitine profiles and flavin levels had been studied and no abnormalities were found.

Mutations in the hRFT2 gene were found in two patients who did not improve on riboflavin: one of the patients with a stable clinical course and the patient in whom treatment was stopped after 1 week for lack of effect. The two patients with a stable condition but no clinical improvement after treatment, and the patient reported to be intolerant of riboflavin supplementation did not demonstrate mutations in *SLC52A1, SLC52A2,* or *SLC52A3.*

## Discussion and future perspectives

This review of the literature demonstrates that - when untreated - BVVL is a severe disorder with a variable but mostly rapid downhill progression, especially in the younger age groups. Outcome is often fatal. As the diagnosis in the group of patients reported before 2010 was based on symptoms only, the disease in these patients may well result from a very diverse etiology.

Since 2010, a much more defined group of patients has been reported. The first publication reported only mutations in the hRFT2 gene and all patients demonstrated abnormalities in acylcarnitine profiles and flavin levels [3]. Since then it has become clear that mutations in all 3 transporters may cause BVVL syndrome [3,8-10].

The abnormalities in the acylcarnitine profiles in the first reported patients are explained by the fact that riboflavin is the precursor of FAD, which acts as an electron acceptor in a number of acyl-CoA dehydrogenation reactions involved in mitochondrial fatty acid oxidation and branched-chain amino acid catabolism [31]. From this first treated group of patients it has become clear that abnormalities in plasma flavin levels and/or plasma acylcarnitine profiles are observed in some, but not in all patients with a hRFT2 or a hRFT3 transporter defect. Importantly, also patients with normal plasma flavin levels and acylcarnitine profiles demonstrated a striking clinical improvement on riboflavin supplementation.

It is now clear that proper diagnosis requires mutation analysis of all three transporter genes. As we reported before, patients will not be detected by newborn screening with abnormal acylcarnitine profiles, probably because of supplementation of flavins from the mother [3]. Because of the striking and often lifesaving effects of riboflavin supplementation it is highly advisable to start treatment immediately without awaiting results of mutation analysis. Two patients in the treated group were treated for a very short period only: in 1 patient treatment was stopped because no clear clinical improvement was seen after 1 week of riboflavin supplementation [7]. However, while in some patients improvement has been demonstrated within days, other patients, and especially more severely affected patients demonstrated a more gradual improvement over a period of more than 12 months. Therefore treating physicians should be advised to continue the trial period for riboflavin supplementation for a longer period. Remarkably, the patient who was treated for 1 week improved in the following months but the relation between the short treatment period and the clinical improvement has remained unclear. Three treated patients who did not improve did however demonstrate a stable course, of which it is not possible to predict whether the course might have been less favorable without the supplementation.

In spite of the success of treatment in this first small group of patients, very little is yet known of the pathophysiology of the disorder, nor of the optimal dose, frequency or mode of administration of the riboflavin supplementation. Further studies into the riboflavin transport by the different transporters are urgently needed.

## Conclusion

Untreated BVVL is a severe and often fatal disorder. In most patients with a proven hRFT defect riboflavin is highly effective and life saving, both in patients with and without a plasma flavin deficiency. The diagnosis can only be made or rejected by mutation analysis of all 3 genes encoding the hRFT family, and treatment should be started immediately without first awaiting results of molecular analysis. Clinical improvement may be rapid or gradual over a period of more than 12 months. We do recommend to rename this disease after its mechanism: Riboflavin transporter deficiency, type 1(hRFT1), 2 (hRFT2) and 3 (hRFT3).

## Competing interests

The authors declare that they have no competing interests.

## Authors’ contributions

AB was involved in design, acquisition and anaylsis of data, drafting of the manuscript. KS was involved in acquisition and analysis of data and drafting of the manuscript. NA was involved in design, analysis of data and critical revision of the manuscript. HW was involved in design, acquisition and analysis of data, drafting of the manuscript. LIJ was involved in design and critical revision of the manuscript. RW was involved in design, analysis of data and critical revision of the manuscript. All authors read and approved the final manuscript.

## Funding

This study was funded by “Stichting Metakids”, The Netherlands.
